# Acute myocardial infarction complicated by purulent pericarditis: A case report

**DOI:** 10.1002/ccr3.6818

**Published:** 2023-01-08

**Authors:** Yamato Tamura, Takehisa Abe

**Affiliations:** ^1^ Department of Cardiovascular Surgery Nara Prefectural Seiwa Medical Center Nara Japan

**Keywords:** methicillin‐sensitive Staphylococcus aureus, purulent pericarditis, sudden cardiac tamponade

## Abstract

A man with persistent dyspnea was brought to our hospital in an emergency. Cardiac catheterization revealed right coronary artery occlusion. The patient went into shock on the second day of treatment due to rapid pericardial effusion. The pericardial fluid was cloudy and non‐bloody, which was judged to indicate purulent pericarditis.

## Graphical abstract

Although bleeding complications are the primary cause of sudden cardiac tamponade in acute myocardial infarction, purulent pericarditis is also a possibility. It is rare but must be diagnosed promptly because it can worsen rapidly by sepsis.

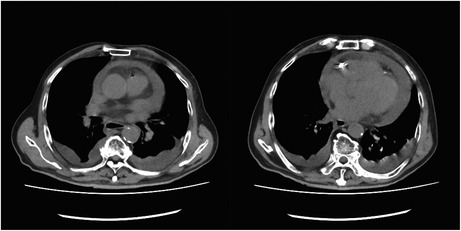

## 1 INTRODUCTION

When sudden cardiac tamponade occurs after acute myocardial infarction, bleeding complications such as post‐acute myocardial rupture, coronary artery hemorrhage, and acute aortic dissection are possible. Our report describes a case of purulent pericarditis complicated by acute myocardial infarction. The reported routes of infection for purulent pericarditis include contamination from open wounds (chest stab wounds or the surgical field), infective endocarditis, pneumonia, subdiaphragmatic spread of infection, and hematogenous spread of a remote infection.

Although hypotension due to pericardial tamponade may occur in purulent pericarditis, it may also arise from septic shock, and appropriate treatment such as drainage and antibiotics should be promptly administered. When cardiac tamponade complicating acute myocardial infarction is encountered, hemorrhagic complications are the primary concern; however, purulent pericarditis also remains a possibility. Purulent pericarditis associated with acute myocardial infarction is rare but may rapidly worsen; thus, it requires prompt diagnosis. To make a prompt and accurate diagnosis, it is necessary to remember that purulent pericarditis is a possible cause of cardiac tamponade after myocardial infarction. The incidence of pericarditis after acute myocardial infarction has decreased with early reperfusion and is reported to range from 4% to 7%. To the best of our knowledge, findings similar to ours have not been reported previously, and we believe that this case report is important to improve patient outcomes in this critical situation.

## 2 CASE PRESENTATION

**FIGURE 1:**
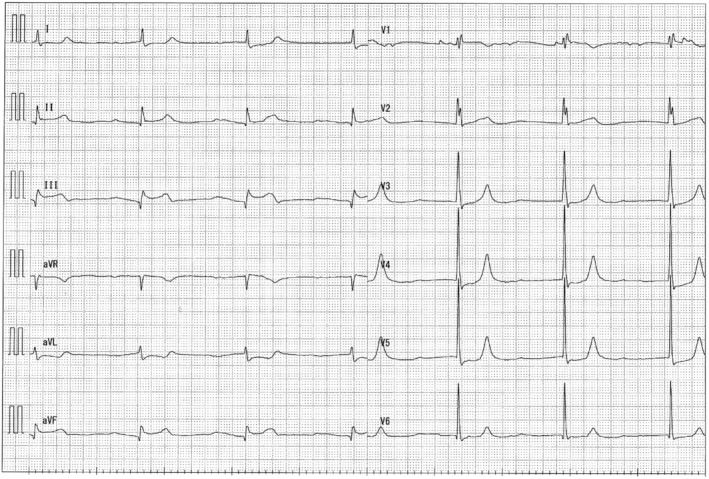
ECG on admission: ST‐segment elevation in leads II, III, and aVF, with a tendency to bradycardia.

**FIGURE 2:**
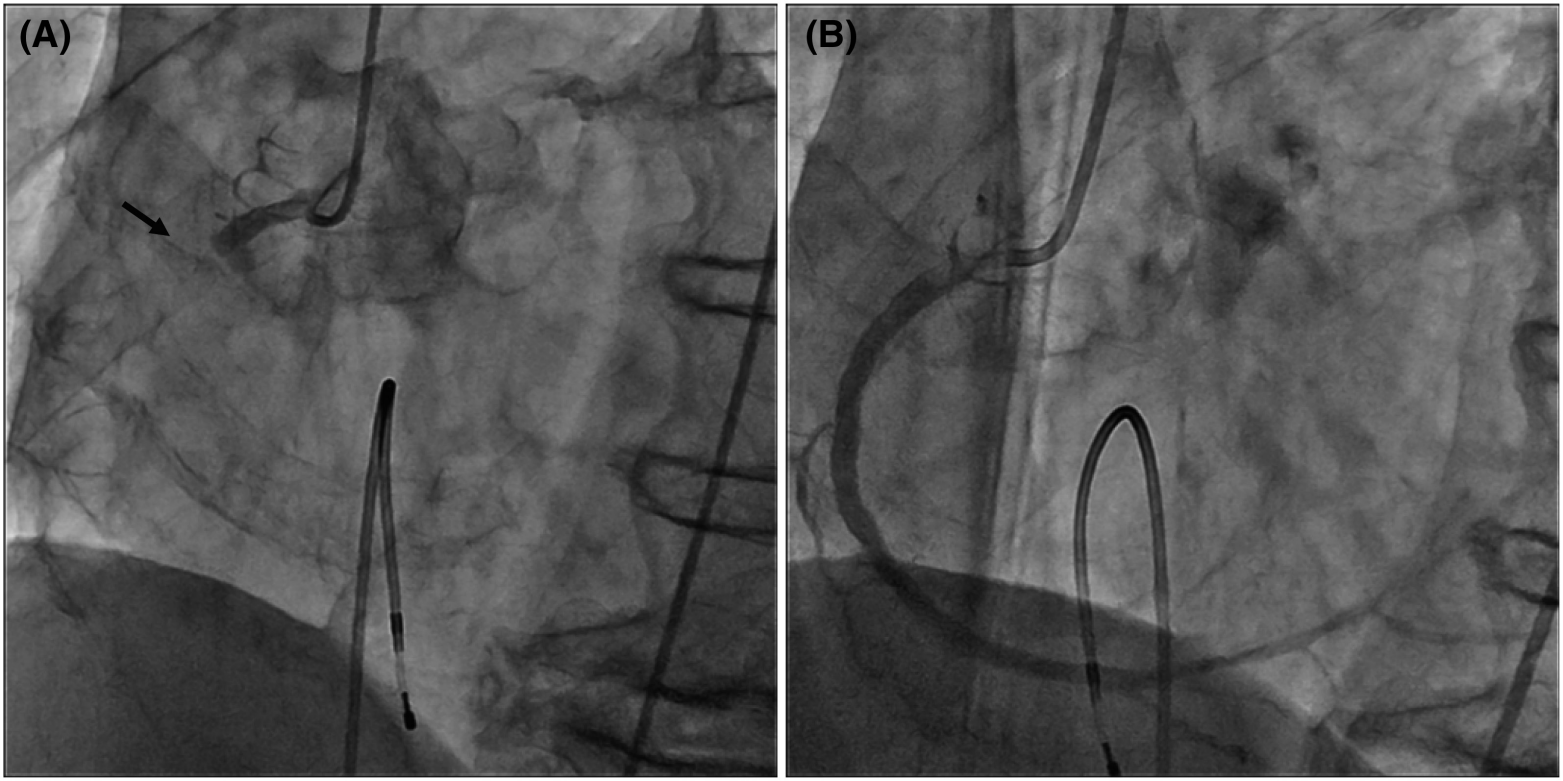
Coronary angiography showing complete occlusion of the right coronary artery (arrow), and revascularization was achieved by catheterization. (A) Coronary artery before treatment. (B) Coronary artery after treatment

A man in his early 80s was rushed to our hospital because he had been experiencing dyspnea since the morning of that day and had shown tendencies toward a worsening condition. On arrival at the hospital, blood pressure was 140/70 mmHg and SpO_2_ was 94% on moist O_2_. He had bradycardia at admission, and electrocardiography (ECG) showed ST‐segment elevation in leads II, III, and aVF (Figure 1). Before admission, there were no symptoms of common cold, such as fever, and no fever was noted at admission. Echocardiography showed the absence of pericardial fluid. The patient had a history of type II diabetes mellitus, hypertension, and dyslipidemia. He had no smoking history. With increased levels of biochemical markers of myocardial injury (CK‐MB 290 ng/ml, troponin I 91.69 ng/ml), the patient was diagnosed with acute myocardial infarction and underwent emergency cardiac catheterization. Before catheterization, the patient was administered aspirin 200 mg and prasugrel hydrochloride 20 mg. Temporary pacing was initiated for bradycardia. The right coronary artery was obstructed, and catheterization was performed (Figure 2). After treatment, the bradycardia improved. Post‐catheterization medications included aspirin, prasugrel hydrochloride, vonoprazan fumarate, atorvastatin calcium hydrate, linagliptin, and empagliflozin. Heparin was continuously infused.

**FIGURE 3:**
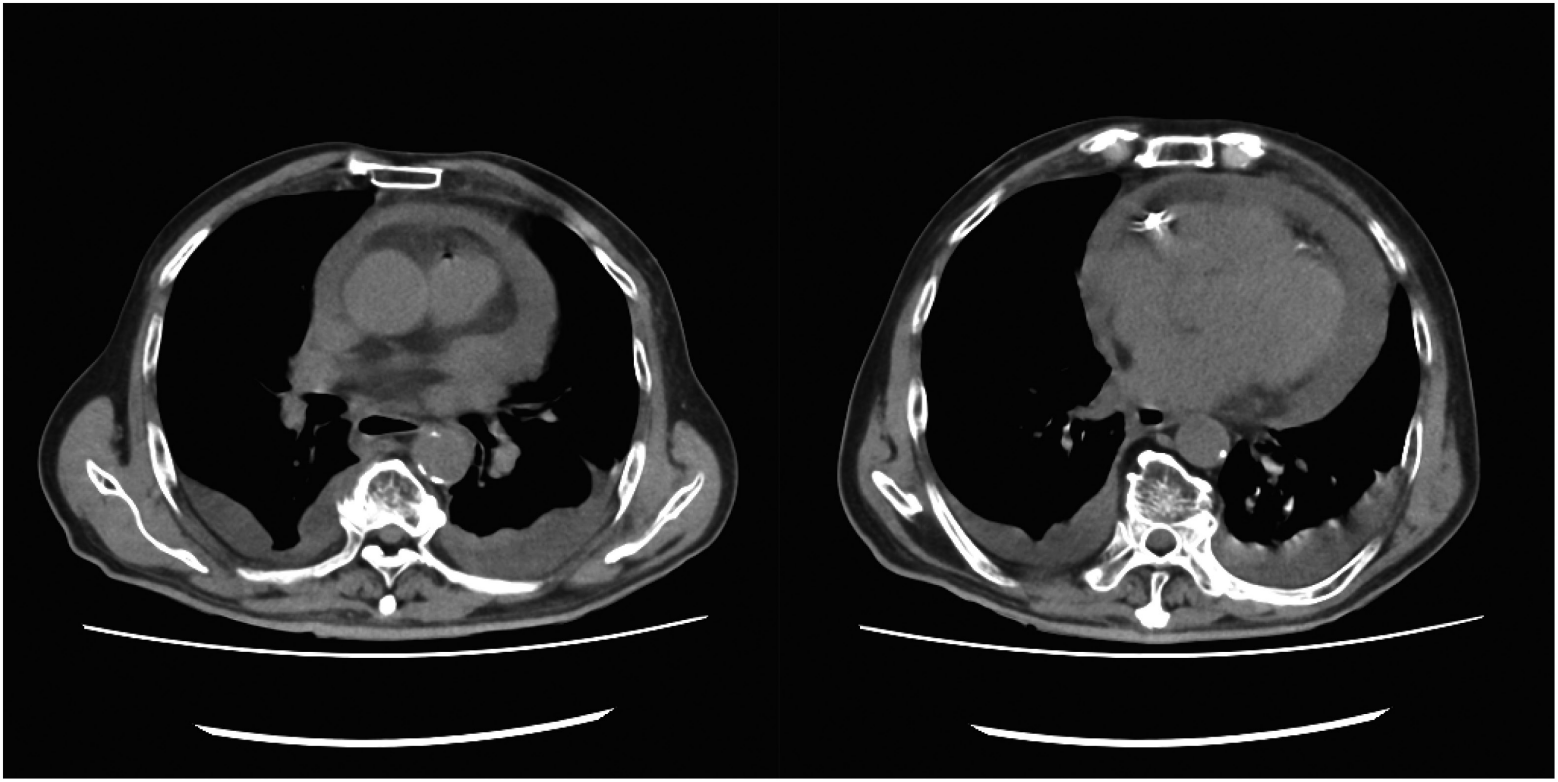
Preoperative computed tomography showing pericardial effusion. There is no evidence of dissection in the ascending aorta.

On the night following treatment, fever and tachypnea were observed, and his respiratory status became poor. There was no vomiting. On the morning of the second day of treatment, the patient's respiratory status further worsened, and echocardiography showed pericardial effusion, which was not present before catheterization. On the second day after the acute myocardial infarction, the patient was hypotensive, blood pressure was 62/48 mmHg, and echocardiography showed cardiac tamponade with additional increase in pericardial fluid. Because of a decrease in blood pressure, shock, and a decreased level of consciousness, we performed endotracheal intubation. Blood tests showed a C‐reactive protein (CRP) level of 30.18 mg/ml and a white blood cell count of 3600/μl. Computed tomography (CT) also showed pericardial effusion (Figure 3), and cardiac rupture after myocardial infarction was suspected. There was no suspicion of aortic dissection.

Because of the possibility of pericardial rupture after an acute myocardial infarction, a median sternotomy, as well as pericardial drainage and hemostasis, were performed. A pericardial hematoma was found in the perfusion zone of the right coronary artery, but there was no obvious pericardial rupture. The pericardial fluid was white and cloudy, and non‐bloody. Infection was suspected, and generous irrigation with a saline solution was applied. A drain was placed, and the chest was closed.

The patient was then placed on a drain and returned to the intensive care unit. After returning to the room, the patient persistently remained in shock and was managed with hypertensive drugs. Postoperative blood tests showed a CRP level of 21.02 mg/ml and a white blood cell count of 1600/μl.

The shock was not alleviated after surgery and continued for a considerable time. A large amount of catecholamine was used, antibiotics were administered, endotoxin adsorption was performed, and the blood pressure was temporarily elevated. However, the blood pressure rapidly decreased the day after surgery, and the patient died. Methicillin‐sensitive *Staphylococcus aureus* (MSSA) was detected in the blood culture and pericardial fluid. Mycobacterium tuberculosis culture was negative.

## 3 DISCUSSION

Pericardial effusion after acute myocardial infarction could lead to pericardial rupture, which occurs in 0.01–0.5% of acute myocardial infarctions and is a fatal complication.^1^ Emergency surgery is required to stop the bleeding to save the patient's life. Other causes of rapid pericardial effusion after acute myocardial infarction include bleeding complications such as aortic dissection and coronary artery hemorrhage.

This case was diagnosed as acute myocardial infarction based on ST changes in ECG leads II, III, and aVF, decreased wall motion of the right ventricle, tendency to bradycardia, and abnormal levels of myocardial enzymes. Coronary angiography also showed occlusion of the right coronary artery. After catheterization, the bradycardia and chest symptoms were relieved. Echocardiography before and after catheterization showed no pericardial effusion.

In the present case, a patient who had an acute myocardial infarction of the right coronary artery developed cardiac tamponade and went into shock on the second day after catheterization. We considered the possibility of cardiac rupture following acute myocardial infarction based on the clinical course of the patient. However, reports of right ventricular rupture are rare,^2^ and we considered the possibility of bleeding due to catheter complications, such as aortic dissection or bleeding from the coronary artery. However, plain CT did not reveal aortic dissection. In the preoperative blood test, CRP was high, indicating the presence of infection. However, the blood pressure was notably low, and we judged that the cardiac tamponade needed to be removed first to improve the patient's hemodynamics. We performed hemostasis through a median incision rather than via a puncture, considering the possibility of hemorrhage. The pericardial fluid was white, cloudy, and non‐bloody, a finding that suggested pericardial effusion due to infection. Subsequently, MSSA was detected in the pericardial fluid and blood cultures. It is possible that the patient developed purulent pericarditis simultaneously or acute myocardial infarction after the onset of purulent pericarditis. However, there were no symptoms of infection before the acute myocardial infarction and no pericardial fluid before or immediately after the catheterization, and the pericardial fluid suddenly accumulated on the second day of treatment. The detection of MSSA suggested the possibility of bacteremia from the skin puncture related to the catheterization and the spread of infection into the pericardial sac. There is a report of myocardial abscess associated with acute myocardial infarction, followed by purulent pericarditis.^3^ However, the presence of a myocardial abscess was not evident in the intraoperative findings.

Although purulent pericarditis is rare in the modern antibiotic era, it is a rapidly progressive condition with a poor prognosis, and early diagnosis and response are necessary to prevent mortality. The routes of infection include chest trauma, surgery, intrathoracic, myocardial, and subdiaphragmatic spread, as well as hematogenous spread from distant sites. In this case, the CT scan showed only gallstones and no obvious infection in the adjacent organs. *Staphylococcus aureus*, *Haemophilus influenzae*, *Neisseria meningitides*, and *Streptococcus pneumoniae* have been reported as causative organisms of purulent pericarditis.^4^, ^5^, ^6^, ^7^, ^8^

Although pericardial infection after catheterization for acute myocardial infarction is rare, it should be considered when the inflammatory response is high, and the intraoperative pericardial fluid is white and cloudy instead of bloody, as in this case. In such a case, the shock state may be caused not only by pericardial tamponade but also by sepsis, and an appropriate response is required for the infection.

Postoperatively, the patient was treated for sepsis with antibiotics and endotoxin adsorption, and although a large amount of catecholamine transiently increased the blood pressure, the state of shock was prolonged, and the patient died.

There have been reports of patients who recovered after pericardiocentesis and antibiotic therapy. Thus, in the future, when a patient with purulent pericarditis presents with shock to the extent that circulation cannot be maintained, it may be necessary to consider not only early antibiotic administration and drainage but also thorough removal of the source of infection, such as continuous irrigation with the chest open. This report details our experience in treating purulent pericarditis complicating an acute myocardial infarction. In the case of sudden pericardial effusion after acute myocardial infarction, cardiac rupture or acute aortic dissection should be considered first; however, if the inflammatory response is high, purulent pericarditis should be included in the differential diagnosis. In such cases, hypotension may not only be due to cardiac tamponade but also to septic shock, and if purulent pericarditis is suspected intraoperatively, thorough treatment of the infection, including drainage and antibiotics, may be necessary.

## AUTHOR CONTRIBUTIONS

**Yamato Tamura:** Data curation; methodology; writing – original draft; writing – review and editing. **Takehisa Abe:** Writing – original draft; writing – review and editing.

## FUNDING INFORMATION

None.

## CONFLICT OF INTEREST

The authors declare no potential conflicts of interest with respect to the research, authorship, and/or publication of this article.

## ETHICAL APPROVAL

Clinicians at our medical center are not required to obtain IRB approval for case reports. The study protocol follows the 2013 Helsinki declaration and its later amendments.

## CONSENT

The patient's family have given written informed consent for the publication of this case report.

## Data Availability

The data that support the findings of this study are available from the corresponding author upon reasonable request.
